# Analysis of Influencing Factors on the Occurrence and Development of Gastric Cancer in High-Incidence Areas of Digestive Tract Tumors Based on High Methylation of GPX3 Gene

**DOI:** 10.1155/2022/3094881

**Published:** 2022-01-12

**Authors:** Jiabei Xie, Lin Fu, Jianmin Zhang

**Affiliations:** Department of Gastroenterology, Henan Provincial People's Hospital, Zhengzhou University People's Hospital, Zhengzhou, Henan 450003, China

## Abstract

Stomach cancer is the second largest cause of cancer-related mortality globally, and it continues to be a reason for worry today. Inhalation of the stomach cancer risk factor *H. pylori* produces large levels of reactive oxygen species (ROS). When combined with glutathione reductase, glutathione peroxidase 3 (GPX3) catalyzes the reduction of hydrogen peroxide and lipid peroxides. To get a better understanding of the GPX3 gene's role in the illness, the researchers used quantitative real-time RT-PCR to examine the gene's expression and regulation in gastric cancer cell lines, original gastric cancer samples, and 45 normal stomach mucosa adjacent to malignancies. According to the research, GPX3 expression was decreased or silenced in eight of nine cancer cell lines and 83 percent of gastric cancer samples (90/108) as compared to normal gastric tissues in the vicinity of the tumor (*P* < 0.0001). It was found that 60 percent of stomach cancer samples exhibited DNA hypermethylation after analyzing the GPX3 promoter (*P*=0.007) (a methylation level of more than 10 percent, as measured by bisulfite pyrosequencing). In stomach tumors, we found a statistically significant reduction in the amount of GPX3 DNA copies (*P* < 0.001). The gene expression of SNU1 and MKN28 cells was restored after treatment with 5-Aza-2′ Deoxycytidine to reduce GPX3 promoter methylation. Genetic and epigenetic alterations lead GPX3 to be dysfunctional in gastric cancer. This indicates that the systems that regulate ROS have been disrupted, and GPX3 may be implicated in the development of gastric cancer, as shown by our results when evaluated alone and in combination.

## 1. Introduction

Gastric cancer is the most common kind of malignancy discovered throughout the globe. In China, the incidence of gastric cancer ranks 2nd among all the malignancies, just below lung cancer. And it is still the 3rd leading cause of cancer-related deaths [1]. There are approximately 1.2 million newly diagnosed cases of gastric cancer worldwide, 40% of which came from China. More than one-half of all deaths worldwide are attributable to stomach cancer among the Chinese population, which has a high prevalence. The endoscopic invasive biopsy is the most common way to diagnose stomach cancer, and it is done while the patient is under anesthesia. Imaging tests are widely used to diagnose stomach cancer, although they have poorer sensitivity and specificity than other established techniques [2]. While early-stage gastric cancer diagnostic capability is limited, tumor markers including carcinoembryonic antigen (CEA), CA19-9, and CA125 are commonly used to evaluate prognosis and therapy effect. There are not many therapy choices for stomach cancer patients since it is usually found at an advanced stage, which is associated with a bad prognosis. This means that early diagnosis of stomach cancer is still a challenge that has to be addressed right away in order to improve the situation. In the glutathione peroxidase (GPX) family, there are eight different enzymes, each of which catalyzes the conversion of glutathione (GSH) to glutathione reductase (GSSG). As a byproduct of cellular oxidative metabolism, ROS are generated [3]. When levels of ROS in the body rise, they have the potential to harm healthy proteins as well as DNA. Only one extracellular antioxidant isoform of selenocysteine, GPX3, is known. It is a member of the GPX family. ROS buildup in tissue cells due to GPX3 inactivation may contribute to cancer growth. ROS damage can be caused by excessive production and accumulation in tissue cells. DNA methylation is one of the most common methods that silence gene expression when it occurs. Different types of malignant tumors have abnormal hypermethylation of the GPX3 promoter and low levels of the GPX3 protein expression, such as thyroid and colorectal tumors [4], Barrett's tumor, prostate and breast cancers, cervical and breast cancers, esophageal cancers, and leukemia and hepatocellular carcinomas. There were varying views on whether or not abnormal GPX3 methylation and expression were present in gastric cancer, but it was generally agreed upon that they were. Quantitative real-time PCR (qRT-PCR) and immunohistochemistry showed by Zhou et al. using pyrosequencing that hypermethylation of GPX3 in gastric cancer among 108 American patients was linked to decreased levels of gene and protein expression (immunofluorescence). The authors in [5] also found that the GPX3 promoter was hyperethylated and that the protein expression of GPX3 was decreased in tissue samples from 22 Korean gastric cancer patients when compared to seven healthy individuals utilizing bisulfite sequencing and immunohistochemistry. According to Min et al., methylation-specific PCR (MSP) and reverse transcription PCR were used to find hypermethylation of GPX3 and low mRNA expression in 10 gastric cancer cell lines. It was shown that in the study conducted on Brazilians (39 cancer tissue samples and 15 normal tissue samples), there was no statistically significant link between GPX3's methylation and the development of gastric cancer. When researchers examined the GPX3 expression in blood and tissue samples from 40 Chinese patients with that of 50 healthy controls, they found that there was no difference. The discrepancies in the aforementioned studies may be ascribed, among other things, to differences in race, location, sample size, and experimental methods. To add to that, studies on the prognostic value of GPX3 methylation have mostly focused on hemophilia and related diseases. One research found that individuals with myelodysplastic syndrome, leukemia transformation, or acute myeloid leukemia who had high levels of GPX3 methylation in their bone marrow had a poor prognosis. Hyperethylated GPX3 was shown to be linked with a worse overall survival rate in people with multiple myeloma, according to Kaiser. However, there is still no convincing evidence linking GPX3 methylation to a better prognosis in patients with gastric cancer at this point. So the goal of this research is to find out whether methylation of the gastric cancer gene GPX3 may be utilized as a prognostic biomarker after radical gastrectomy in 118 Chinese patients with gastric cancer who underwent a quantitative MSP methylation (qMSP) test. The remaining section of the paper can be organized as follows.  Section 1 depicts the basic introduction to gastric cancer and GPX3. The related existing methodologies are depicted in Section 2. The problem definition was depicted in Section 3. The technique used for analyzing lung cancer is defined in Section 4, while the findings and explanations for the study are in Section 5. Section 5 ultimately summarizes the paper.

## 2. Related Works

More and more study indicates that reactive oxygen species (ROS) play a role in stomach cancer growth and progression through a molecular mechanism. It is possible to regulate ROS via the elimination of free radicals by glutathione peroxides (GPx), the essential enzymes that maintain ROS homeostasis in vivo (RO). This article summarized studies on the molecular mechanism and function of different GPxs, including their varied roles in the incidence, development, and progression of gastric cancer, to better understand gastric cancer etiology and therapeutic possibilities. As a result of those investigations, it offered a possible path for future research on the treatment of stomach cancer [6]. The epigenetic mechanisms involved in gastric cancer, such as DNA methylation, histone modifications, and the chromatin remodeling complex, are summarized. As long as research into stomach cancer continues, we will not be far from mapping an epigenome code. Finally, as previously said, epigenetic therapy may be made accessible in the not too far future [7]. GPX7, on the other hand, was shown to be downregulated in all seven cancer cell lines examined and in almost half of all human gastric cancer cases (22/45). In contrast to all seven gastric cancer cell lines and 56% (25/45) of gastric cancer samples, only 13% (6/45) of normal samples showed DNA hypermethylation (>10% methylation level) of the GPX7 promoter (*P* < 0.0001). The treatment of AGS and SNU1 cells with 5-aza-2′-deoxycytidine significantly demethylated the GPX7 promoter and restored GPX7 expression. According to in vitro studies, reconstituted GPX7 inhibited the development of stomach cancer cells in two-dimensional and three-dimensional organotypic cell culture models [8]. The goal of this research was to develop a prognostic model for GC patients using genes linked to lipid metabolism [9]. Epigenetic and genetic mechanisms in esophageal cancer cells were studied by researchers [10]. The molecular landscape of gastric cancer has improved thanks to the use of next-generation sequencing (NGS), and new classification methods for molecular heterogeneity have been proposed. This paves the way for new, more focused therapies [11]. This field covers a wide range of topics, from the tumor microenvironment to germline mutations, and everything in between. Stomach cancer risk may be decreased by ascorbic acid, photochemical, and the body's natural antioxidant processes. There may be a connection between tobacco smoking and decreased levels of gastric juice ascorbate, as well as achlorhydria and chronic atrophic gastritis caused by *H. pylori* infection and the bacteria's release of CagA. In gastric cytoprotection, ascorbic acid's antioxidant properties may help prevent stomach cancer by increasing stomach glutathione and vitamin E levels, reducing toxic effects of nitrosodimethylamines and other heterocyclic amines, and decreasing the growth of the H. pylori bacteria that can cause it. Ascorbic acid may also help reduce the risk of gastric cancer through these same mechanisms. Effectiveness is related to the virulence of *H. pylori* strain, particularly to CagA expression, in this cytoprotection study. Currently, researchers are looking into the role of vitamin C in stomach cancer epigenetic reprogramming. Stomach cancer may be exacerbated by a lack of vitamin C and other nutrients. A cure for stomach cancer may be found by eliminating *H. pylori* and restoring vitamin C synthesis by the stomach's epithelium [12]. With TRAMP mice with early-stage prostate cancer, researchers looked at the relationship between dietary animal fat and antioxidant GPx3 expression. There were no differences in the mortality rates among the two groups of animals that were given a high-fat (45 percent of Kcal fat) or a control diet after five or ten weeks of feeding (10 percent Kcal fat). The histopathological score increased with age and with a high-fat diet [13]. Researchers are looking at how epigenetic changes affect the body's ability to produce cancer and the potential regulators that may help prevent it. It is important to note that many papers in the fields of epidemiology, biomarkers, genetics, gastroenterology, and endoscopic treatment explain the molecular and biochemical alterations that occur as BE progresses. We systematize the information from these many areas into a complete overview [14]. Numerous genes that had been significantly elevated in cancer tissue were now decreased in adjacent no tumor tissue after treatment with 5-aza-2′-deoxycytidine (5-Aza-dC). To investigate the expression and methylation status of potential candidate genes in five human ESCC cell lines, we used methylation-specific polymerase chain reaction (PCR) and bisulfite genomic sequencing in conjunction with two human immortalized epithelial cell lines, primary ESCC tumor tissues, and adjacent nontumor tissues for comparison, as well as immunohistochemistry [15]. In this article, the author discusses how the demethylating agent 5′-aza-2′-deoxycytidine, which has been tweaked to increase methylation of the MLH1 gene, sensitizes colon cancer cells to the chemotherapy drug 5-fluorouracil. Patients with DDR defects are an especially good candidate for synthetic lethality because of the possible advantages. PARP inhibitors, the most powerful anticancer medicines now available, work best on BRCA-deficient cancer cells. The article [16] proposed DNA methylation in carcinoma. In [17], genome-wide methylation levels in Northeast Indian blood samples were assessed in the present study using an Infineon 450k array and blood samples from the community. Later, the methylation information was combined with the rest of the information. An integrome analysis was used to build a gene network with enriched pathways and also generated a list of genes with hypermethylated or hypoethylated promoters and inversely related expression, respectively. There are 23 genes in the integrome network that are associated with tumor growth and metastasis, including cell adhesion, integrin signaling, cytoskeleton, and the extracellular matrix architecture. During this time, 19 genes were unregulated (SEMG2, CD97, CTNND2, CADM3, and OMD), four genes were downregulated (PTK2, RND1, RND3, and UBL3), and their promoters were hypoethylated (which indicates that they were upregulated), and four genes (PTK2, RND1, RND3, and UBL3) were upregulated (which indicates they were hypoethylated). COL1A1, TAC3, SERPINA4, TNFSF13B, and IL221RA2 were the top-ranking genes on the Methylation Efficiency Index (MEI). In [18], there is a creation of ESCC ATLAS (ESCC Genomic, Epigenetic, Transcriptomic, and Proteomic Atlas), a collection of carefully chosen information on ESCC-related genes from published studies.

## 3. Proposed Work

### 3.1. Adenoviral Vector Design

Adenoviruses are increasingly being employed in clinical studies as vectors for the delivery of transgenes for gene therapy, vaccination vectors, and as vectors for oncolytic virotherapy. Many properties of adenoviruses make them useful in this context. Many studies have been done on viral biology; adenovirus genomes are simple to alter and can contain a lot of extra DNA. They can infect both proliferating and nondividing cells, which makes them useful for a wide range of research and development applications. Some elements of vaccine development benefit from the humoral and cellular immune responses that are induced by these agents. Immunogenicity of adenovirus vectors, on the other hand, may lead to toxicity, which reduces the efficiency of transgene delivery. Vectors for adenoviruses lack crucial viral genes and so are replication-deficient. E1A and E1B are often missing from first-generation vectors, while second-generation vectors lack even more nonessential areas, such as E2 and E4 transcription units, to improve the capacity for exogenous DNA. Vectors that are reliant on helpers, or “gutted vectors,” include just the viral sequences necessary for DNA replication, such as the inverted terminal repeats that act as DNA replication “origin” sequences and viral packing sequences. These adenovirus vectors can clone more cells per unit of time than the first and second generations combined. Helper-dependent adenovirus vectors must be grown in the presence of a complementary helper adenovirus that delivers the missing early and late genes necessary for the generation of virus particles. Several approaches have been developed to remove the helper-dependent adenovirus vector from the helper adenovirus and to package just the vector DNA. Transgene delivery efficacy and transgene expression length may be limited by the host's innate immune response and preexisting immunity to adenovirus vectors. When used in conjunction with immunosuppressive medicines, some of these host reactions may be mitigated although adenovirus vector modifications have also been utilized to circumvent the host response and boost transgene delivery effectiveness. It is possible to cover immunogenic epitopes and inhibit immune detection by covalently modifying the adenovirus capsid with synthetic polymers like polyethylene glycol. Including receptor-specific targeting ligands or moieties that affect the host's innate immune responses in modified capsid regions may also help with immune evasion. Because of the prevalence of preexisting anti-HAdV-5 antibodies, it may be difficult to employ HAdV-5-based vectors. Preexisting immunity may be minimized by using chimeric adenovirus vectors, which substitute parts of a HAdV-5-based vector with those from a different kind. Two further ways to bypass previous immunity are using vectors based solely on other HAdV types or nonhuman AdVs. HAdV-5-based vectors have a variety of alterations that improve replication, oncolysis, and tumor immunosurveillance. Newer HAdV genotypes have also been produced as innovative vectors to solve the difficulties associated with HAdV-5-based constructions. To help patients with disorders caused by mutant versions of a gene, researchers have been experimenting with adenovirus vectors as delivery platforms for transgenes. There have been clinical studies using adenovirus vectors to try to fix faults in cystic fibrosis, muscular dystrophy, coagulation problems, and metabolic defects such as ornithine transcarbamylase deficiency, among other things. adenovirus vectors' ability to induce an immune response inspired researchers to employ them in the development of many different types of vaccination, most notably for the prevention of malaria and HIV, among other illnesses. Newly designed single-cycle adenovirus vectors have recently been produced to circumvent the dangers associated with fully replication-competent Ad vectors and may be used as vaccine platforms. Recent advances in the development of adenovirus vectors have enabled the display of foreign immunogenic peptides on the viral capsid surface by inserting peptides containing epitopes of interest in capsid proteins, hexon, fiber, and protein IX. For the treatment of diseases such as melanomas, head and neck squamous cell carcinomas, non-small-cell lung cancer, prostate cancer, and numerous others, adenovirus vectors have been modified for use. Angiogenesis may be inhibited by expressing tumor antigens or immunomodulatory proteins; tumor cell death can be enhanced by expressing tumor suppressor proteins like *p*53 or factors capable of suppressing angiogenesis. The delivery of an enzyme to selectively activate a medication capable of killing a designated cell is another strategy. One example of this is the conversion of ganciclovir to an active form by a viral thymidine kinase vector-delivered. To combat cancer, researchers have looked at using replication-competent adenovirus vectors. These vectors can destroy a cell directly throughout the viral life cycle, making them ideal for cancer therapy. These methods, like others that employ adenovirus vectors, rely on figuring out how oncolytic viruses may be modified to bypass host defenses while still selectively targeting cancerous cells.

The schematic representation of the suggested methodology was illustrated in Table 1.

### 3.2. Declaration of Ethical Principles

**Figure 1:**
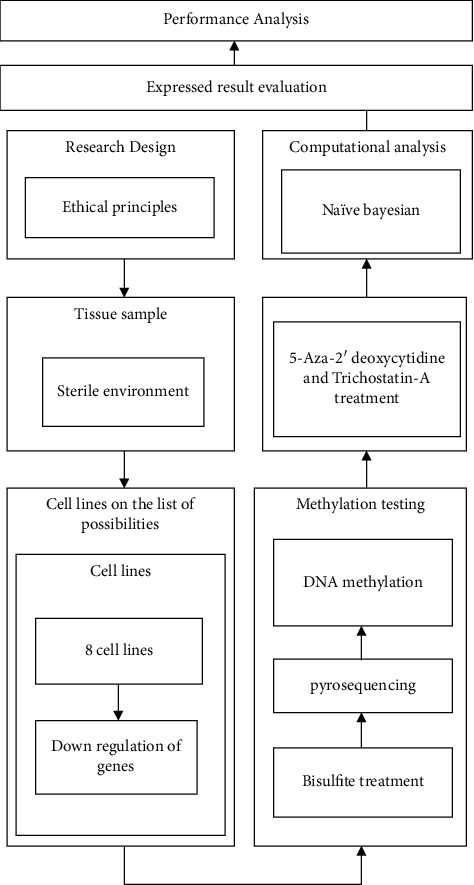
Schematic representation of the suggested methodology.

The National Cancer Institute's CHTN (Cooperative Human Tissue Network) provided deidentified human tissue samples from the Vanderbilt University pathology archives in Nashville, Tennessee (CHTN). The Institutional Review Board at Vanderbilt University Medical Center authorized the use of specimens in this research. Tissue samples were obtained from all patients who signed consent forms and had their moles surgically removed. In this study, every single sample of tissue was obtained from tissues that were discarded once a diagnosis was established (Figure 1).

### 3.3. The Tissue Samples That Were Collected

All of our tissues were supplied by the National Cancer Center China (NCCC). The Institutional Review Board authorized the use of the university's historical tissue collection in this study. Almost all of the materials used in this research were coded and culled from tissues that would have been wasted or destroyed if they had not been coded and culled. This is something that must be remembered at all times. The participants' ages varied from 38 to 64, with a median of 64. Participants in the research ranged in age from 38 to 87. The patients were 38 to 87 years old, with a median age of 64 (there are 70 males and 38 women). All of the tumors have been histologically verified. Stages I through IV depicted intestinal tumors and widespread cancers. The well-differentiated (WD) stomach adenocarcinomas were separated from the less well-differentiated (WD) stomach adenocarcinomas (PD). Scientists considered the stomach mucosa epithelial tissues next to tumors “normal” since they showed no signs of neoplastic transformation.

### 3.4. Select Cell Lines from the List of Possible Outcomes

The China Type Culture Collection (https://www.wipo.int/budapest/en/idadb/details.jsp?id=5827) and Riken (Ibaraki, Japan; http://www.brc.riken.go.jp/lab/cell/english) each have nine gastric cancer cell lines available: MKN28, MKN45, MKN75, and KATO3. Cells from the AGS cell line were kept alive for further study by supplementing the medium with 10% fetal bovine serum and antibiotics. We cultivated all cell lines at 37°C in a humidified environment with 5% CO_2_.

### 3.5. Quantitative Real-Time Reverse Transcriptase PCR

Total RNA was extracted from the samples using the RNase Mini-kit, and the results were analyzed using a quantitative real-time PCR test (qRT-PCR). Single-stranded cDNA was synthesized using the iScript cDNA Synthesis Kit and utilized in the following study. This is the first time that the GPX3 gene has been shown to be expressed in 153 primary human samples, which included 108 samples of gastric carcinoma and 45 samples of normal gastric mucosa next to the stomach malignancies. Samples were taken from individuals with stomach cancer and from healthy gastric mucosa around tumors, according to the researchers. For persons who have 24 malignancies, tumor-matched normal mucosa from the same people may be utilized to ensure that they get successful treatment. In order to make the forward and reverse primers, we utilized Primer 3 (http://frodo.wi.mit.edu/) (http://frodo.wi.mit.edu/). The threshold cycle number was determined using a Bio-Rad iCycler (version 3.0), and the results were analyzed using the qRT-PCR technique described below. The answers must be repeated three times to reach the threshold values. HPRT1 reference gene findings were compared with those of the GPX3 gene, which exhibited little variation in both normal and tumor samples examined and is thus deemed a reliable and stable reference gene for RT-PCR (forward 5′-GGAAAGGGTGTT TATTCCTCA-3′, reverse 5′-TCCCAGGTCAGC AAAGAA-3′). We used the equation 2(Rt–Et)/2(Rn–En) to calculate the formula. All primary gastric cancer samples had a relative mRNA expression of less than 0.5, indicating that the gene was downregulated.

### 3.6. The Use of Bisulfite Treatment of DNA in conjunction with Pyrosequencing for Analysis

First, a DNase Tissue Kit was used to separate the DNA from the tissue samples (Qiagen). An EZ DNA Methylation-Gold Kit was used to bisulfite-modify the DNA of cell lines and tissues according to the manufacturer's instructions. CpG island of GPX3 promoter identified by USC Norris CpG island search engine (http://www.uscnorris.com) has been previously mentioned. The pyrosequencing primers for this experiment were designed using the PSQ Assay Design Software (Biotage, Uppsala, Sweden). It was expected to be a basic process. The idea was to devise a process that anybody could use. The sequencing primer GGGAGGGGTAAGT was utilized for all three types of probes in the study, functioning as a forward, reverse, and sequencing primer pair. A polymerase chain reaction required the primers and Platinum PCR SuperMix High Fidelity Enzyme Mix to amplify the appropriate promoter region (PCR). An amplification assay using PCR was carried out on a 40 ng modified DNA sample (Nitrogen, Carlsbad, CA). PCR gel electrophoresis was needed to verify the results and exclude primer dimer formation as a potential source of error. As instructed by the manufacturer, quantitative pyrosequencing analysis of the required PCR data was performed using a Biotage PyroMark MD System (Biotage). The data was analyzed using Pyro Q-CpG 1.0.9. Hypermethylation of the GPX3 promoter was found to be low in hypermethylated samples; thus, this cutoff value was used to identify them. Between cancer and control samples, we performed statistical analysis to see whether there were any changes in DNA methylation at CpG sites.

### 3.7. Aza-2′ Deoxycytidine and Trichostatin-A Are Used in the Treatment

The effects of DNA hypermethylation on GPX3 transcriptional regulation were examined using the gastric cancer cell lines SNU1 and MKN28. For long-term survival in addition to antibiotics, SNU1 and MKN28 cells were cultivated in DMEM with 10% fetal bovine serum (FBS). Low-density seeded cells were treated with 5-Aza-2′ promoter deoxycytidine (5-Aza) for 24 hours before being treated with 300 nM Trichostatin-A for another 24 hours (Wako, Osaka, Japan). All of the RNA and DNA in the samples were extracted and purified using the Qiagen Tissue Kits. Pyrosequencing, a sophisticated technique, allowed us to determine how much DNA methylation was present at each of the GPX3 promoter's CpG nucleotides. The transcript levels of GPX3 were determined using the previously published technique.

### 3.8. Immunofluorescence

SNU1 was treated with 5-Aza and the protein expression was examined by immunofluorescence labeling against GPX3. They were permeabilized for 2 minutes with Triton X-100 in 0.1 percent sodium citrate and rinsed in fresh 4 percent par formaldehyde after 72 hours of treatment with 5-Aza (Sigma-Aldrich) or 300 nM Trichostatin-A (TSA, Wako). 20 minutes were spent at room temperature before it was collected and treated with 10% normal goat serum. First, cells were treated for 45 minutes at room temperature with an Alexa Fluor 488-conjugated primary goat anti-rabbit antibody before being incubated overnight at 4°C with a secondary goat anti-rabbit antibody as described above (11000). DAPI, a fluorescence-sensitive glue, was utilized to mount the slides in the fluorescence microscope.

### 3.9. A Quantitative PCR Assay for the Determination of DNA Copy Numbers Was Developed (qPCR)

The qPCR amplifications were carried out using a Bio-Rad iCycler to determine relative DNA copy counts in this study (Bio-Rad). The iCycler software version 3.0 was used to determine the PCR reaction threshold cycle number in a total volume of 20 l. The PCR tests were carried out using 40 ng template DNA and 20l PCR reactions as the reaction volume. Using iCycler 3.0, we were able to determine the threshold cycle number. Afterward, the information was compared to predictions. Primer 3 (http://frodo.wi.mit.edu/) was used to make the primers, which may be found at http://frodo.wi.mit.edu. Primers for GPX3 genomic DNA were designed using the 5′-CCCCTTCAGTAGGGCCTAAG-3′ sequence. The 5′-TTCTTCAGGACCAGGACCAGGACCAGGACCAC reverse primer was selected for GPX3. For the GPX3 forward primer, the following sequences were incorporated in the genomic DNA: The primers used in this research were supplied by IDT (Integrated DNA Technologies) (Coralville, Iowa). To discover the ideal threshold values, scientists conducted three different tests (CT). The average CTs for actin and GAPDH were compared to ensure consistency. There was minimal difference in the results across all of the CT scans for the normal and tumor samples. By analyzing mRNA expression with qRT-PCR, the DNA copy number was calculated and normalized to the average of 10 healthy blood DNA samples (2 copies, equal to copy number ratio 1.0). The number of DNA copies dropped when the relative cutoff ratio was less than 0.5.

### 3.10. Research on the Design and Development Adenoviral Vectors for the Expression of GPX3

The GPX3 coding sequence was transferred to the plasmid using the Platinum PCR SuperMix High Fidelity after being amplified with the Flag-tag and cloned into pACCMV.pLpA. According to a research published in the journal Nature Communications, the findings were made public. In order to generate and disperse recombinant adenoviral particles expressing the GPX3 gene, which were transected with the pJM17 vector, the pACCMV.pLpA-GPX3 plasmid had been utilized.

### 3.11. Statistical Analysis

The naïve Bayesian technique is also one of the machine learning techniques in the supervised method. This technique is the probabilistic classifier which is given by Thomas Bayes. This classification technique has the existence or nonexistence of the features which is independent of the existence. It believes the file is a bag of words that has the probability of methylation in the data and its location is to be independent of each other. If we assume that the file is FL and the class is CS, so the probability will be evaluated as follows:

(1) $$\begin{array}{c} \text{Prob}\left(\frac{\text{CS}}{\text{FL}}\right)=\frac{\text{Prob}\left(\text{FL}/\text{CS}\right)\text{Prob}\left(\text{CS}\right)}{\text{Prob}\left(\text{FL}\right)}. \end{array}$$

Then, the probability sentiment is to be evaluated as follows:

(2) $$\begin{array}{c} \text{Prob}\left(\frac{\text{methylation}}{\text{normal}}\right)=\frac{\text{Prob}\left(\text{methylation}/\text{normal}\right)\text{Prob}\left(\text{methylation}\right)}{\text{Prob}\left(\text{sentence}\right)}. \end{array}$$

## 4. Performance Analysis

**Figure 2:**
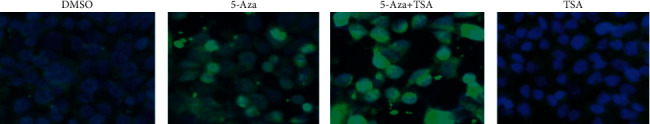
5-Aza treatment restored GPX3 gene expression.

As a result of treating these cell lines with 5-Aza, GPX3 mRNA levels returned to normal in both the SNU1 (A) and MKN28 (B) strains (Figure 2). Neither the restoration of GPX3 expression nor the alteration of methylation levels was affected by TSA treatment despite the fact that TSA treatment restored 5-Aza-induced gene expression in a substantial cumulative manner. Following 5-Aza therapy, there was a reduction in GPX3 methylation as well as an increase in gene expression. We used an anti-GPX3 antibody to conduct an immunofluorescence test in SNU1 cells to see whether 5-Aza therapy also restored GPX3 protein levels. Figure 2(c) indicates that the 5-Aza and 5-Aza-TSA treatments significantly increased the GPX3 green immunofluorescence signal when compared to the DMSO control, indicating that these drugs may restore GPX3 protein expression in these cells (Figure 2).

**Figure 3:**
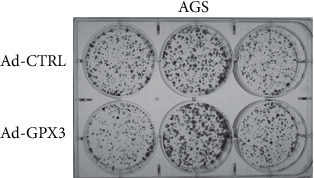
GPX3 reconstitution.

**Figure 4:**
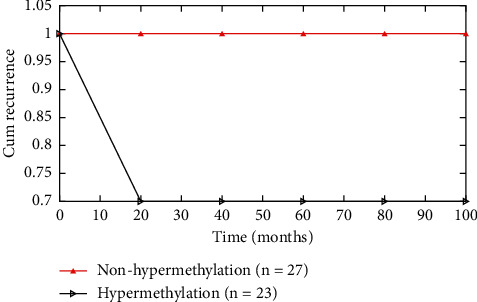
Time versus Cum recurrence.

In the Methods section, 5-Aza and TSA are used to treat SNU1 and MKN28 cancer cells. Figure 2 depicts the suggested implementation. The left panel displays the GPX3 expression ratios in SNU1 cells A and B after adjusting for HPRT (MKN28 cells). A comparison pane on the right shows samples with similar levels of DNA methylation. Anti-GPX3 antibody staining of SNU1 cells is as follows. 5-Aza-2′ deoxycytidine compounds include 5-Aza-2′ deoxycytidine and 5-Aza-2′ deoxycytidine, which may be found in a broad range of chemical forms (Figure 3). To see this, we used 5-Aza to demethylate the GPX3 promoter in SNU1 and the 5-Aza treatment resulted in restored GPX3 mRNA levels (Figure 2) [19]. There was no difference in the methylation levels after TSA treatment alone. TSA treatment, however, exhibited a substantial additive impact in restoring gene expression after 5-Aza administration. After 5-Aza, TSA therapy resulted in increased gene expression and a reduction in GPX3 methylation. We used an anti-GPX3 antibody in an immunofluorescence experiment in SNU1 cells to see whether 5-Aza therapy also restored GPX3 protein levels. For example, as shown in Figure 4(c), the GPX3 green immunofluorescence signal increased significantly following 5-Aza and 5-Aza-TSA treatments in comparison to the DMSO control. This suggests GPX3 protein expression may be restored by 5-Aza and 5-Aza-TSA treatments.

We hypothesized that GPX3 would act as a tumor suppressor in gastric cancer in the same way. On AGS and MKN28 cell lines, we used soft agar colony formation tests to help restore GPX3 gene expression. Tumor suppressor function may be comparable in gastric cancer, according to our hypothesis. We used AGS and MKN28 cell lines to reestablish GPX3 gene expression and conducted colony formation and soft agar colony formation experiments on those cell lines, respectively. Interestingly, the number of colonies formed by GPX3-expressing AGS cells and control cells did not vary significantly. Soft agar assays in AGS and MKN28 cells produced similar findings (Figure 3). The number of colonies generated by AGS cells expressing GPX3 was the same as that of control cells, which was unexpected.

In order to assess the prognostic significance of GPX3 hypermethylation, a naïve Bayes curve was utilized with a cutoff value of 37% of the methylated reference. The average recurrence time was 23.40 months for the whole sample (Figure 4).

**Figure 5:**
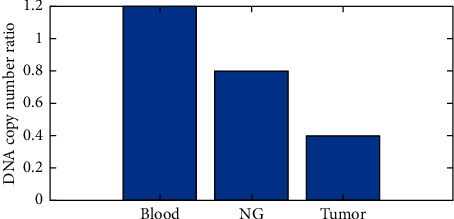
DNA copy number ratio.

When researchers used a quantitative polymerase chain reaction (qPCR) to assess the GPX3 copy number (Figure 5), they also looked at beta-actin and GAPDH copy numbers in the same samples. This result was checked to see whether it was still valid by comparing it to the copy number of GPX3 found in 10 additional healthy blood samples (copy number ratio equals 1.0). Normal tissues around gastrointestinal cancer showed fewer GPX3 copies than the malignancy itself (Table 2).

**Figure 6:**
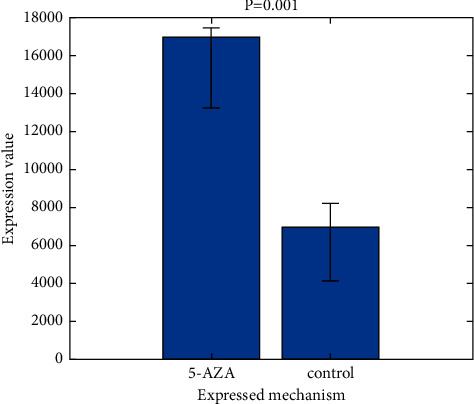
Expressed mechanism versus expressed value.

When it came to 48 cases of gastric cancer, researchers discovered an antiparallel relationship between GPX3 methylation and the levels of the ontogeny's messenger RNA (mRNA) (Figure 6). The expressed value of *P* should be 0.001.

**Figure 7:**
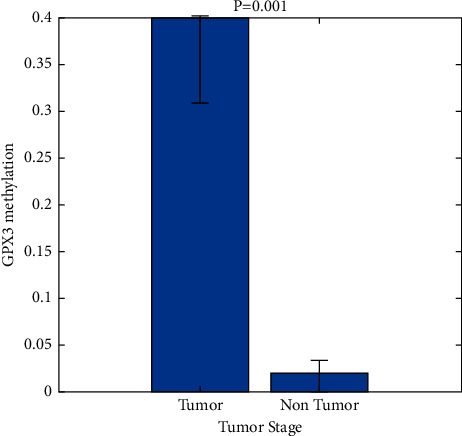
Tumor stage GPX3 methylation.

A patient's prognosis for stomach cancer was evaluated by looking at GPX3 methylation (Figure 7). We determined the GPX3 methylation threshold (PMR = 3.79%) in malignant tissues and divided 118 patients with gastric cancer into two groups: those with hypermethylation and those without it. Sixty-one individuals died during follow-up, and 23 had cancer recurrence seven years following surgery, according to the results. Following up with patients for an average of 29 months was common (max: 83 months, min: 1 month). The whole group had an average recurrence time of 23.40 months (max: 82 months, min: 0.5 months).

## 5. Conclusions

A new research shows that GPX3 inhibits the growth and spread of stomach cancer. Tumor suppression in Gpx3 was absent in two additional gastric cancer cell lines (AGS and MKN28), indicating that Gpx3 may have a distinct function in other organs. However, we found that the migratory ability of GPX3-expressing cells was reduced in our scratch wound-healing experiment. According to these results, GPX3 downregulation and hypermethylation are essential for lymph node metastatic development. When GPX3 is administered to mice, cancer metastasis is prevented, but further research is needed to determine how this is accomplished. Patients with gastric cancer who have a promoter or copy number decrease inactivating the GPX3 gene are at greater risk of lymph node metastases. Stomach cancer patients experience oxidative DNA damage because the tumor's antioxidant defenses are compromised. More study is needed to determine if GPX3 contributes to the onset, progression, or metastasis of gastric cancer.

## Figures and Tables

**Table 1:** Gp*x*3 functional characteristics.

Species	Exons	Regulatory elements	Chromosomal location
Gp*x*3	Human	5	AP-1
	Mouse	5	TCDD (mRNA and protein), c-maf

**Table 2:** Percentage of methylation.

Research samples	DNA copy number to total number of DNA molecules	Methylation in terms of percentage	mRNA expression ratio
Normal sample	1.00	5.0	1.0
AGS sample	0.50	3.0	0.1
MKN28 sample	0.60	91.00	0.0
MKN45 sample	0.30	92.000	0.0
MKN75 sample	0.60	91.000	0.0
KATO3 sample	0.20	88.00	0.0
SNU1 sample	0.40	94.00	0.0
SNU5 sample	0.80	4.00	0.9
SNU16 sample	0.30	6.00	0.1
RF1 sample	0.60	24.00	0.0

## Data Availability

The datasets used and/or analyzed during the present study can be available from the corresponding author if needed.
